# ProbeAlign: incorporating high-throughput sequencing-based structure probing information into ncRNA homology search

**DOI:** 10.1186/1471-2105-15-S9-S15

**Published:** 2014-09-10

**Authors:** Ping Ge, Cuncong Zhong, Shaojie Zhang

**Affiliations:** 1 Department of Electrical Engineering and Computer Science, University of Central Florida, Orlando, FL 32816, USA; 2 Present address: J. Craig Venter Institute, 4120 Torrey Pines Road, La Jolla, CA 92037, USA

**Keywords:** RNA structure probing, High-throughput sequencing, RNA secondary structure, Homology search

## Abstract

**Background:**

Recent advances in RNA structure probing technologies, including the ones based on high-throughput sequencing, have improved the accuracy of thermodynamic folding with quantitative nucleotide-resolution structural information.

**Results:**

In this paper, we present a novel approach, ProbeAlign, to incorporate the reactivities from high-throughput RNA structure probing into ncRNA homology search for functional annotation. To reduce the overhead of structure alignment on large-scale data, the specific pairing patterns in the query sequences are ignored. On the other hand, the partial structural information of the target sequences embedded in probing data is retrieved to guide the alignment. Thus the structure alignment problem is transformed into a sequence alignment problem with additional reactivity information. The benchmark results show that the prediction accuracy of ProbeAlign outperforms filter-based CMsearch with high computational efficiency. The application of ProbeAlign to the FragSeq data, which is based on genome-wide structure probing, has demonstrated its capability to search ncRNAs in a large-scale dataset from high-throughput sequencing.

**Conclusions:**

By incorporating high-throughput sequencing-based structure probing information, ProbeAlign can improve the accuracy and efficiency of ncRNA homology search. It is a promising tool for ncRNA functional annotation on genome-wide datasets.

**Availability:**

The source code of ProbeAlign is available at http://genome.ucf.edu/ProbeAlign.

## Background

The non-coding RNAs (ncRNAs) play various vital roles in the biological systems [1-3], such as gene-expression regulation [4], catalysis [5], signal recognition [6], and ribosomal RNA modification [7]. Given the facts that most of the human genome (approximately 62% [8] to 93% [9]) is transcribed [10] while only a small fraction of it (about 3%) actually codes for proteins, it is tempting to hypothesize that the ncRNAs contribute enormously to the complex and elegant regulatory networks in human and other multicellular organisms. Therefore, fully understanding any biological system is impossible without the thorough research on the ncRNAs in it. However, annotating ncRNAs is more difficult than proteins, because ncRNAs with divergent sequences may fold into conserved secondary structures, and still perform similar biological functions. In this sense, secondary structure conservation is used as a better evidence for functional conservation than sequence similarity when conducting comparative ncRNA analysis.

Annotating the ncRNA secondary structure is a prerequisite for comparative ncRNA structural analysis. However, determining ncRNA secondary structure is a non-trivial task. The performance of the existing computational methods (such as mfold [11], RNAfold [12], and RNAstructure [13]) for predicting secondary structure from a single ncRNA sequence is not satisfying, especially for long ncRNA sequences [14]. Although the prediction accuracy can be improved with evolutionary information from multiple sequence alignments [15-19], such information is not always available for every genome of interest. On the other hand, genome-wide annotation of known ncRNA families by homology search still appears as an open problem for lacking efficient and accurate computational pipelines. For example, the latest release of the widely used software CMsearch has significantly improved the computational efficiency of its previous versions [20]. However, it still would take about 3 hours to annotate the 1 Gbp chicken genome with known Rfam [21] families on a 100-CPU cluster even with filters and MPI applied [20]. The sensitivity of CMsearch reaches a plateau at ~87% without filters, indicating intrinsic difficulty in detecting ncRNAs with diverse sequences. The difficulty of ncRNA annotation is partly due to the computational overhead of structure alignment, and partly due to the low information content given by the secondary structures [22].

Recent advances in massive parallel sequencing make genome-wide probing of ncRNA secondary structures possible. Examples of technologies in this category include, but not limited to, PARS [23], FragSeq [24], and SHAPE-seq [25] (SHAPE-seq has not been applied in genome-wide study). With further improvements, such techniques are becoming more powerful for understanding the *in vitro *[26,27], or even *in vivo *ncRNA structrome [28]. The information received from a typical genome-wide ncRNA secondary structure probing experiment is the *reactivity *for each site. As the probing reagents, such as 1M7 [25,29,30], DMS [28], or nuclease [24], are chosen to preferentially attack the paired/unpaired regions, the experimentally determined reactivity can be used to assess the probability of whether a specific site is paired. The reactivities can then be transformed into pseudo-energy terms [30,31], and be incorporated into existing RNA-folding algorithms to predict the optimal secondary structure that is compatible with both the RNA free energy models and the observed reactivity pattern. When the reactivity information derived from SHAPE technology [29] was incorporated, the 16s rRNA structure prediction accuracy was lifted from 47% to 97% [30]. This success implies great potential in using the structure probing information in other comparative genome-wide ncRNA analysis approaches.

Therefore, it is possible to improve the ncRNA annotation by incorporating the high-throughput RNA secondary structure probing information. First, the computational efficiency can be promoted by only focusing on transcribed regions revealed by the read-mapping pattern as used in standard RNA-seq experiments. In addition, the experimentally defined structural information can be used to reduce the search space of the alignment algorithms and lead to the development of a more efficient one. Meanwhile, we can also expect to improve the annotation accuracy because the experimentally determined structural information reflects the real RNA structures, and is much more accurate than the *in silico *predictions.

Here, we present a novel ncRNA annotation algorithm called ProbeAlign, which, to the best of our knowledge, is the first algorithm that considers high-throughput RNA structure probing information for the purpose of genome-wide ncRNA annotation. To make ProbeAlign feasible for large-scale sequencing data, the specific pairing relationships between bases in the query structures are discarded to achieve *O*(*n*^2^) time complexity. On the other hand, with the usage of structure probing data, the partial structural aspects of target sequences are introduced into the algorithm. Therefore, ProbeAlign tackles the homology search problem from another perspective with the support of new technologies. The benchmark results show that the prediction of ProbeAlign outperforms filter-based CMsearch with a shorter running time. Last but not least, the application of ProbeAlign to FragSeq data, which was generated by high-throughput sequencing-based RNA structure probing technology, shows its capability of analyzing genome-wide datasets.

The rest of the paper is organized as follows: in the Methods section, we discuss the core algorithm of ProbeAlign and how to estimate the *p*-values for the alignment scores. In the Results section, we describe benchmark results, parameters optimization and an application of our algorithm to FragSeq data. In the Discussion section, we summarize our existing works, and propose possible directions for future research.

## Methods

### Algorithm

ProbeAlign identifies the homologous ncRNAs in a profile-based search manner. The profile is generated by using the multiple sequence alignment of a given ncRNA family. The aligned columns formed by a majority of gap are excluded in the search profile. In addition, the consensus structure of the family is considered as the structural information of the profile. The targets of search are usually the genomic or transcriptomic sequences with experimentally determined reactivities. In the latest implementation of ProbeAlign, higher reactivity of a site indicates higher chance of being unpaired, and vice versa.

Assume the alphabet of RNA sequences is {A, C, G, U, X}, in which X represents all unknown nucleotides. First we denote the query of an ncRNA family as *Q *= {*P, S*}, where *P *is the sequence profile of the family and *S *is the pairing pattern in the corresponding consensus structure. Let the length of the profile be *n*, then *P *= 〈*p*_1_*, p*_2_*, . . . , p_n_*〉 and *S *= 〈*s*_1_*, s*_2_, . . . , *s_n_*〉. Here, $p_{i}=\left[v_{i}^{A},v_{i}^{C},v_{i}^{G},v_{i}^{U},v_{i}^{X},v_{i}^{-}\right]$, which is a vector that contains the frequency of the nucleotides and gap at site *i. s_i _*is a boolean value indicating whether site *i *is paired in the consensus structure or not (0 means *i *is paired and vice versa). Note that the specific pairing relationship between sites in *P *is not considered in *S*, which is similar to the folded-BLAST [32]. For target *T *of length *m*, denote *B *= 〈*b*_1_*, b*_2_*, . . . , b_m_*〉 as the genomic sequence and *R *= 〈*r*_1_*, r*_2_*, . . . , r_m_*〉 as the observed reactivities. Denote *D_i,j_, I_i,j_, M_i,j _*as the optimal alignment scores for deleting, inserting and matching a column in the search profile, respectively. They can be computed using the following recursive functions:

(1) $$\begin{array}{c} D_{i,j}=max\left\{M_{i-1,j}+\varepsilon_{0}+\varepsilon_{e},D_{i-1,j}+\varepsilon_{e}\right\},\\ I_{i,j}=max\left\{M_{i,j-1}+\varepsilon_{0}+\varepsilon_{e},I_{i,j-1}+\varepsilon_{e}\right\},\\ M_{i,j}=max\left\{D_{i,j},I_{i,j},M_{i-1,j-1}+\alpha \times \tau \left(s_{i},r_{j}\right)+\beta \times \sigma \left(p_{i},\;b_{j}\right)\right\}. \end{array}$$

Here, ε_0 _and ε*_e _*are the gap open penalty and the gap extension penalty, respectively. In our implementation, a "semi-global alignment" setting [33] is adopted. Therefore, these three functions are initialized with: *M*_0,0 _= 0, *M*_*i*,0 _= ε_0 _+ *i × *ε*_e_, M*_0*,j *_= 0, and *D*_0*,j *_= *I*_*i*,0_= −*∞. τ *and *σ *are functions to assess the structural and the sequence similarities between the queries and the targets, respectively. *α *and *β *are weights assigned to these two types of similarities.

The sequence similarity between the query profile and the target sequence is computed using the following formula:

(2) $$\sigma \left(p_{i},b_{j}\right)=\underset{x\text{ϵ}\left\{\text{A},\;\text{C},\;\text{G},\;\text{U},\;\text{X},\;-\right\}}{\sum }v_{i}^{x}\times \text{m}(x,b_{j}).$$

where m(*x, y*) is the substitution score between nucleotides *x *and *y*.

The general function to compute structural similarity is as follows:

(3) $$\tau \left(s_{i},\;r_{j}\right)=\left\{\begin{array}{cc} 0 & \text{if }r_{j}\;\text{is not defined,}\\ f\left(s_{i},r_{j}\right) & \text{otherwise}\text{.} \end{array}\right)$$

Given the reactivity *r_j_, p*(*π_j_| r_j _*) is computed to compare the structural aspect of *b_j _*with *s_i_. π_j _*is a random variable and *π_j _*∈ {0, 1}, 0 means *b_j _*is paired and 1 means *b_j _*is not paired. According to the Bayes' theorem, the probability can be computed as:

(4) $$p\left(\pi_{j}\text{|}r_{j}\right)=\frac{p\left(r_{j}\text{|}\pi_{j}\right)\times p\left(\pi_{j}\right)}{{{\text{Σ}}_{\pi_{j}}}_{}p\left(r_{j}\text{|}\pi_{j}\right)\times p\left(\pi_{j}\right)}.$$

The probabilities *p*(*r|π *= 0) and *p*(*r|π *= 1) can be inferred from the reactivities retrieved from the RNAs with known secondary structures [34]. To simplify the computation, we assume *p*(*π *= 0) is equal to *p*(*π *= 1) and then define the function *f *as:

(5) $$\begin{array}{cc} f\left(s_{i},r_{j}\right) & =\text{log}p\left(\pi_{j}=s_{i}\text{|}r_{j}\right)-\text{log}p\left(\pi_{j}\neq s_{i}\text{|}r_{j}\right)\\ & =\text{log}p\left(r_{j}\text{|}\pi_{j}=s_{i}\right)-\text{log}p(r_{j}|\pi_{j}\neq s_{i}). \end{array}$$

Note that the probability *p*(*r|π*) varies among different probing techniques. Even for one protocol, the reactivity distributions may be different due to the distinct computational methods for transferring the chemical signals from biological experiments. Therefore, it may be hard to apply Equation 5 on some techniques whose statistical properties have not been studied yet. To overcome this limitation and make the implementation of ProbeAlign easy to use, a simplified scoring function is proposed:

(6) $$f\left(s_{i},r_{j}\right)=\left\{\begin{array}{cc} 1 & \text{if}\left(r_{j}>r_{c}\;\text{and}\;s_{i}=1\right)\;\text{or}\;\text{(}r_{j}<r_{c}\;\text{and}\;s_{i}=0\text{),}\\ -1 & \text{otherwise}\text{.} \end{array}\right)$$

In Equation 6, *r_c _*is a cutoff value which is used to annotate the structural aspects of targets. Any site that has higher reactivity than *r_c _*is deemed as unpaired, and vice versa (*r_j _> r_c _*⇒ *p*(*π_j _*= 1*|r_j _*) = 1; *r_j _<*= *r_c _*⇒ *p*(*π_j _*= 0*|r_j _*) = 1). We have compared two different types of structural similarity functions by taking SHAPE protocol as an example. The benchmark results show that the optimal performance of those two functions is comparable. Therefore, the simplified scoring function is practical for universal usage, while the protocol-specific scoring function may be a better option if the reactivity distribution is known.

**Figure 1:**
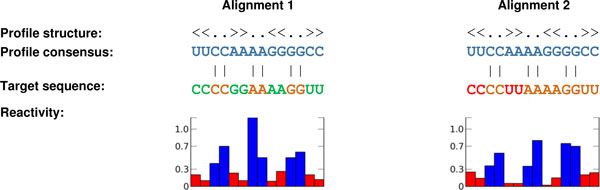
**Two alignments with different structure consistency scores**. The reactivities with red color are higher than *r_c_*, while the reactivites with blue color are less than *r_c_*. In Alignment 2, the red letters can not form canonical base pairs. The green letters in Alignment 1 can form canonical base pairs.

The above described dynamic programming algorithm computes the optimal alignment between the query profile and the target sequence with the consideration of both structural and sequence similarity. After alignment, traceback is performed to check the base pairing consistency between the query structure and the target. Bonus scores are assigned to the possible pairing bases. Such additional information can be used to detect potential false positive hits that have high alignment scores but low structural consistency with the query. For example, in Figure 1, two alignment scores are the same. However, the target in alignment 1 is more conserved with the query, compared with the target in alignment 2, because the red letters can form canonical pairs, while the green ones can not. Structure consistency scores can help us distinguish these two targets.

### P-value estimation

A robust scheme for evaluating the statistical significance of prediction results is important for the homology search applications. However, what statistical distribution the ncRNA alignment scores should follow is still unclear. In this case, we simulated the ProbeAlign scores by searching 106 Rfam families (as defined by the Infernal RMARK3 dataset [20]) against five artificial sequences, whose GC content ranging from 30% to 70%. Each artificial sequence was constructed by concatenating random RNA sequences generated by GenRGenS [35] with a simple context-free grammar [36]. The secondary structure of each random sequence was predicted by mfold [11]. The corresponding reactivities of the secondary structure were simulated based on the SHAPE technology [34].

**Figure 2:**
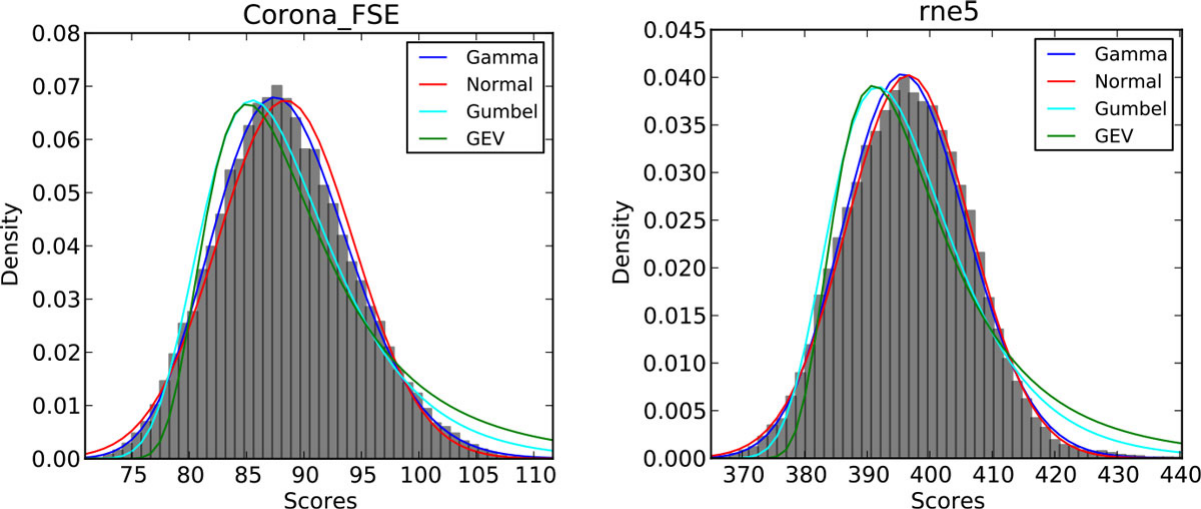
**Fitting of the alignment score distributions for Corona_FSE and rne5 families**.

We fitted the ProbeAlign score density for each Rfam family with four different distributions: the normal, Gumbel, GEV (Generalized Extreme Value), and Gamma distributions. The goodness of fitting was measured with K-S test (Kolmogorov-Smirnov test). The fitting results on the five artificial sequences show that the ProbeAlign scores follow the Gamma distribution for most of Rfam families. Take the fitting on the artificial sequence with 50% GC content as an example. Out of 106 tested families, 103 families fit best with the Gamma distribution, and the other three families (bicoid_3, OLE, and rne5) fit best with the normal distribution. The score distribution fitting of the Corona_FSE family (which follows Gamma) and the rne5 family (which follows normal) is shown in Figure 2. It is clear that even rne5 fits better with the normal distribution, the Gamma distribution also fit the ProbeAlign score distribution well. So the Gamma distribution was chosen to evaluate the *p*-values in ProbeAlign.

## Results

### Benchmarks

In this section, we will compare the performance of ProbeAlign and CMsearch using the RMARK3 benchmark dataset. This dataset contains 106 families, and each family has a training alignment and a test set. The training alignments were employed to generate queries for both tools. The sequences in the test sets were concatenated together and served as the target in the experiments. The corresponding reactivities of the target were simulated based on the SHAPE technology [34]. To make the comparison between ProbeAlign and CMsearch fair, for each family, we kept the number of predictions of these two programs the same. A server machine with 4 Xeon i7 CPUs (2.4 GHz) and 128 GB RAM was used for the benchmarking and subsequent experiments under single core configuration.

CMsearch adopts the covariance model to query against the target sequences to detect RNA homologs. The recent release of CMsearch is coupled with Hidden Markov Model (HMM)-based filters to improve its computational efficiency [20]. In the following experiments, CMsearch will be invoked with default setting, which means the filters are coupled and the default parameters are used. For ProbeAlign, the weights for the structural and sequence similarity, *α *and *β*, were set to 0.7 and 2.6, respectively. The simplified scoring function for structural similarity was used as default in the benchmarks. According to the property of the SHAPE reactivity data [30], *r_c _*was set to 0.3. A detailed discussion of parameter selection will be presented in the following section.

The synthesized target contains 780 ncRNA sequences (160,390 bps) from the RMARK3 dataset. It takes 2.13 minutes CPU time for ProbeAlign to finish the search while it takes 6.85 minutes CPU time for CMsearch. Such improvement is expected, since ProbeAlign adopts an *O*(*mn*) algorithm, while the core algorithm of CMsearch is from *O*(*mn*^1.3^) to *O*(*mn*^2.4^) [37], for a query with *n *sites and a target with *m *bases. In terms of prediction accuracy, the overall TP/FP ratio of CMsearch is 632/292, while that of ProbeAlign is 653/271. Of the 106 ncRNA families in RMARK3, ProbeAlign generates different prediction results with CMsearch in 27 families. Table 1 shows the performance difference of ProbeAlign and CMsearch on those families.

**Table 1:** Summary of the results of ProbeAlign and CMsearch on the RMARK3 dataset.

Rfam ID	Name	Identity	# Tests	# Predictions	CMsearch		ProbeAlign	
					TP	FP	TP	FP
RF00005	tRNA	44%	20	61	10	51	**16**	45
RF00007	U12	61%	7	8	**7**	1	6	2
RF00013	6S	43%	38	24	21	3	**24**	0
RF00017	SRP_euk_arch	46%	21	24	19	5	**21**	3
RF00020	U5	52%	22	23	19	4	**22**	1
RF00023	tmRNA	48%	59	59	**58**	1	57	2
RF00028	Intron_gpl	34%	20	5	4	1	**5**	0
RF00030	RNase_MRP	44%	28	36	16	20	**22**	14
RF00066	U7	62%	2	1	**1**	0	0	1
RF00080	yybP-ykoY	46%	13	13	**13**	0	10	3
RF00104	mir-10	58%	2	1	0	1	**1**	0
RF00114	S15	61%	8	11	**8**	3	7	4
RF00140	Alpha_RBS	65%	3	4	1	3	**3**	1
RF00165	Corona_pk3	68%	1	4	0	4	**1**	3
RF00177	SSU_rRNA_5	49%	13	17	12	5	**13**	4
RF00230	T-box	46%	48	50	46	4	**47**	3
RF00504	Glycine	50%	14	14	**14**	0	13	1
RF00515	PyrR	46%	29	38	25	13	**28**	10
RF00534	SgrS	48%	3	2	0	2	**1**	1
RF00548	U11	57%	8	11	**7**	4	5	6
RF00640	MIR167_1	53%	10	9	**8**	1	7	2
RF00645	MIR169_2	52%	21	21	**21**	0	20	1
RF00661	mir-31	57%	3	3	2	1	**3**	0
RF01052	Arthropod_7SK	65%	2	3	0	3	**2**	1
RF01066	6C	67%	1	2	**1**	1	0	2
RF01069	purD	56%	8	9	**8**	1	7	2
RF01296	snoU85	62%	2	6	1	5	**2**	4
	Overall		406	459	322	137	**343**	116

Only the families with different results between the two programs are shown in the table.

**Figure 3:**
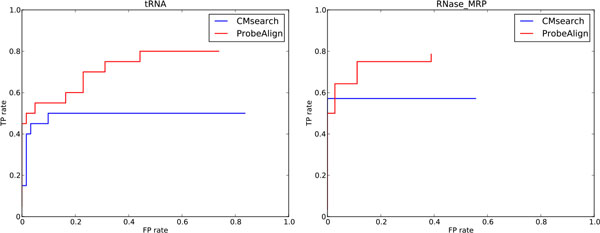
**ROC plots for the performance of CMsearch and ProbeAlign in searching tRNA and RNase_MRP**. CMsearch is invoked with the default parameters and filters. TP rate is computed by dividing the number of TP predictions by the size of the training set. FP rate is computed by dividing the number of FP predictions by the total number of all predictions.

**Figure 4:**
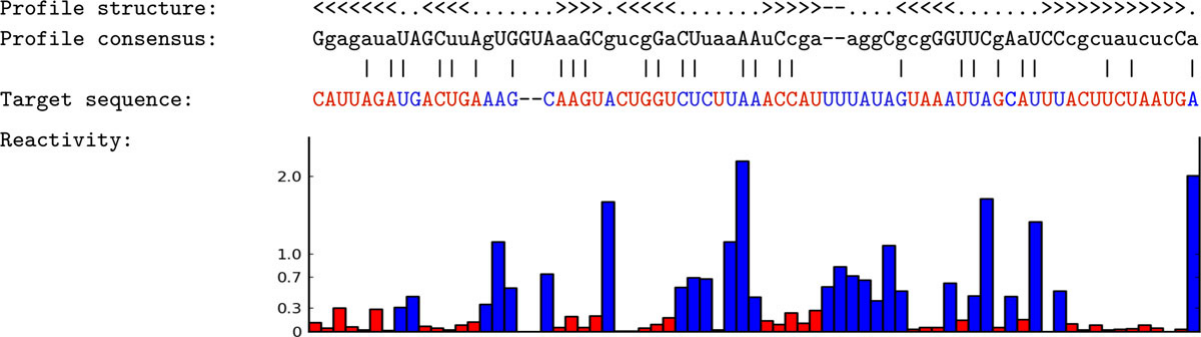
**An alignment generated by ProbeAlign between the tRNA query profile and a target tRNA sequence**. The accession ID of the target RNA sequence is AY632242.1/10-80. The red color in target sequence and bars indicates the sites with low reactive scores (*< r_c_*). The blue color indicates the sites with high reactive scores (*> r_c_*).

The search results for the tRNA and RNase_MRP families, whose ROC curves are shown in Figure 3, clearly demonstrate the advantage of using the probing information to detect remote homologous sequences. The sequence identities for these two families are 46% and 47%, which make it challenging for HMM-based filters to find the tested RNAs. Note that it would be possible for CMsearch to predict more low sequence identity hits by disabling the filters, but it would dramatically (by 10,000-fold) increase the running time [20]. On the other hand, when the probing information is considered, the high structural similarity is able to compensate the low sequence similarity, and lift the ranking of ncRNA sequences that are difficult to be detected by CMsearch. Figure 4 shows that a tRNA homolog (Accession ID: AY632242.1/10-80) missed by CMsearch was identified by ProbeAlign. Of 71 sites in the profile of the training set, 13 sites have frequencies less than 12.5% and 22 sites have frequencies between 12.5% and 25%, which prevents the HMM filters to retrieve some tRNAs.

### Optimizing the structure and sequence similarity weights

In the ProbeAlign algorithm, the parameters *α *and *β *indicate the weights for the structural and sequence similarity, and control how the two types of information are incorporated into the dynamic programming algorithm. The setting of these parameters should reflect how well the probing data would represent the actual secondary structure, as well as which information is more important in defining a specific ncRNA family. Ideally, the setting of the parameters should be family-specific to satisfy the structure and the sequence conservation patterns. However, it is tedious to define a set of parameters for each search profile, and more importantly, the overly tweaked parameters for the under-represented families would even bias the search. In this case, it is expected to apply a set of universal parameters for all families.

**Figure 5:**
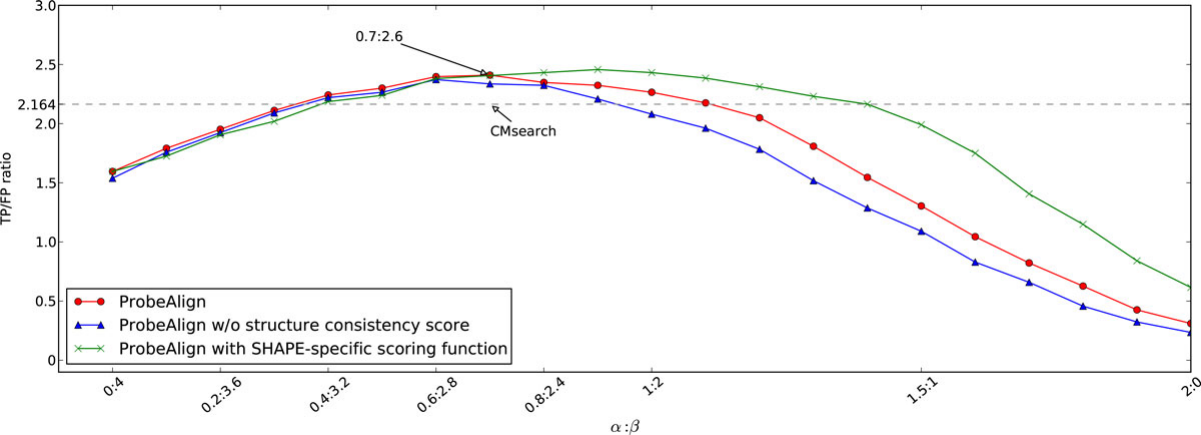
**Performance of ProbeAlign with different structure and sequence similarity weights**. The TP/FP ratio of CMsearch, 2.164, is represented as a dash line in the figure.

Three experiments have been conducted to analyze the effect of *α *and *β *on the performance of ProbeAlign by using the RMARK3 dataset. The value of *α *varied from 0 to 2 with an increasing step of 0.1, while the value of *β *varied from 4 to 0 with a decreasing step of -0.2. In the first experiment, we investigated the performance of ProbeAlign with the default setting under different combinations of *α *and *β*. In the second experiment, we excluded the structure consistency score to investigate its contribution to the overall performance. In the third experiment, the prediction was based upon the SHAPE-specific scoring function for structural similarity. Figure 5 shows the performance of ProbeAlign in these three experiments. For the first experiment (Figure 5, red line), the optimal performance is achieved at *α *= 0.7 and *β *= 2.6, which is then taken as the default setting for the algorithm. For the second experiment (Figure 5, blue line), the optimal performance is achieved at *α *= 0.6 and *β *= 2.8. The performance of ProbeAlign is higher than that without considering the structure consistency score. Such improvement is more significant when the structural weight is higher. Therefore structure consistency score is an effective way of improving the overall performance. In the last experiment, we adopted the SHAPE-specific scoring function to evaluate the structural similarity between the Rfam families and the target sequence. We can see that the optimal performance for the SHAPE-specific function (Figure 5, green line) and the default simplified function (Figure 5, red line) is comparable: 656/268 at *α *= 0.9 and *β *= 2.2 for the SHAPE-specific scoring function; 653/271 at *α *= 0.7 and *β *= 2.6 for original simplified scoring function. The performance difference is increased when raising the ratio of *α*/*β*. Therefore, the protocol-specific scoring function may be a better choice if the underlying reactivity distribution is known. The implementation of ProbeAlign allows users to decide which scoring function they use.

### High-throughput sequencing-based RNA structure probing data

FragSeq (fragmentation sequencing) is a genome-wide RNA structure probing technique that has been applied to study the mouse nuclear transcriptome [24]. The RNA secondary structures in the KH2 undifferentiated mouse embryonic stem cells (undiff) and neural precursors cells (d5np) were probed. By analyzing nine snRNA families (U1, U2, U3, U4, U5, U6, U8, U11, and U12) in the mouse genome, the paper shows a good accordance between the probing data and the real secondary structures. New secondary structures have also been proposed to three other snRNA families (U15, U22, and U97), showing the ability of FragSeq to discover novel ncRNA transcripts and their secondary structures.

We used ProbeAlign with default setting to search the nine snRNAs families against the FragSeq data to demonstrate its utility on experimentally determined reactivities. We were only interested in the genomic regions that were transcribed, i.e. being covered by more than 4 sequenced reads. There are 18,388 regions (32.2 Mbps) for the undiff cell line and 17,007 regions (29.0 Mbps) for the d5np cell line. The reactivities for these regions were computed using FragSeq v0.0.1, a supplementary software for the probing protocol [24]. Because FragSeq is a different technology than SHAPE, *r_c _*was adjusted to 0.5 from 0.3. All other parameters remained the same as in the benchmark. A universal *p*-value cutoff (0.01) was set for all searches. The running time for the undiff dataset was 30.20 minutes CPU time, and for the d5np dataset was 26.97 minutes CPU time. During the analysis of the predicted results, we found some reads were mapped onto repeat regions in the genome. Those hits were removed by using Repbase database [38]. The final search results are summarized in Table 2. U11 and U12 have no record in Repbase. Only 17 and 21 U4 records in Repbase are covered by the transcribed regions of d5np and undiff cell lines, and all of them are top ranked in the results of ProbeAlign. The corresponding sequences with their locations on the genome can be downloaded at http://genome.ucf.edu/ProbeAlign.

**Table 2:** Summary of the prediction results by ProbeAlign on the FragSeq data.

	RF00003	RF00004	RF00012	RF00015	RF00020	RF00026	RF00096	RF00548	RF00007
	U1	U2	U3	U4	U5	U6	U8	U11	U12
d5np	46(46)	18(18)	11(11)	243(17)	12(12)	120(117)	2(2)	1	4
undiff	46(46)	19(19)	11(11)	302(21)	12(12)	146(134)	2(2)	1	4

The numbers in the brackets show that how many predictions by ProbeAlign are recorded in Repbase.

One interesting observation from the ProbeAlign search results is that the transcription of U4 and U6 snRNA families are more active in undiff cells than in d5np cells. It is not surprising to see the potential correlation between the U4 and U6 transcription level, as they have been proposed to interact with each other in splicing control. In fact, it is hypothesized that they can bind with each other due to a long complementary sequence between them [39]. Recent experiments show that the snRNAs in un-proliferated stem cells have higher expression than in proliferated cells [40]. The observation is explained by the snRNAs playing an important role in ribosome biogenesis, cellular proliferation and pre-mRNA splicing [41]. From the ProbeAlign search results, we can further conclude that not only the expression level of the snRNA is higher in un-proliferated cells, there are actually more U4 and U6 snRNA genes being transcribed in un-proliferated stem cells.

## Discussion and conclusions

In this article, we have proposed a novel algorithm, ProbeAlign, for incorporating high-throughput sequencing-based RNA structure probing data into ncRNA homology search. To our knowledge, this is the first application of structure probing information to RNA functional annotation. This integration makes the accuracy of ProbeAlign even higher than the CMsearch tool, especially for ncRNA homologs with low sequence identity. In addition, the time complexity of the algorithm is *O*(*n*^2^), which is feasible for handling genome-wide datasets.

ProbeAlign itself can also act as a filter for more detailed downstream alignment algorithms. Considering both ProbeAlign and the HMM filters in CMsearch being *O*(*n*2) time complexity algorithms, they should have comparable time efficiency if similarly optimized. It is clear that ProbeAlign guarantees higher sensitivity and specificity. In this case, ProbeAlign can be coupled with more accurate alignment algorithms such as CMsearch itself, or other structure-sequence alignment algorithms such as FastR [42], PFastR [43], and RSEARCH [44]. We are also developing a new structure-sequence alignment algorithm that takes into account the probing information, which can also be used as the downstream detailed alignment after ProbeAlign screening.

In conclusion, we present here an accurate and efficient RNA homology search algorithm, ProbeAlign, which incorporates the high-throughput sequencing-based RNA structure probing information. With the increasing requirement of genome-wide ncRNA annotation, we anticipate that more RNA transcripts, and their secondary structures and functionalities, will be annotated by using ProbeAlign.

## Competing interests

The authors declare that they have no competing interests.

## Authors' contributions

SZ contributed with the conception of the research. PG, CZ, and SZ designed the algorithm. PG implemented the algorithm and conducted the experiments. PG, CZ, and SZ wrote the manuscript. All authors read and approved the final manuscript.

## Declarations

Publication of this article was supported by the National Institute of General Medical Sciences of the National Institutes of Health, USA (R01GM102515).

This article has been published as part of BMC Bioinformatics Volume 15 Supplement 9, 2014: Proceedings of the Fourth Annual RECOMB Satellite Workshop on Massively Parallel Sequencing (RECOMB-Seq 2014). The full contents of the supplement are available online at http://www.biomedcentral.com/bmcbioinformatics/supplements/15/S9.
